# Tumor regression and safe distance of distal margin after neoadjuvant therapy for rectal cancer

**DOI:** 10.3389/fonc.2024.1375334

**Published:** 2024-04-04

**Authors:** Guilin Yu, Huanyu Chi, Guohua Zhao, Yue Wang

**Affiliations:** ^1^Department of General Surgery, Cancer Hospital of China Medical University, Liaoning Cancer Hospital & Institute, Shenyang, China; ^2^The Second Clinical College, Dalian Medical University, Dalian, China

**Keywords:** rectal tumor, neoadjuvant therapy, distal resection margin, tumor regression, safe distance

## Abstract

Neoadjuvant therapy has been widely employed in the treatment of rectal cancer, demonstrating its utility in reducing tumor volume, downstaging tumors, and improving patient prognosis. It has become the standard preoperative treatment modality for locally advanced rectal cancer. However, the efficacy of neoadjuvant therapy varies significantly among patients, with notable differences in tumor regression outcomes. In some cases, patients exhibit substantial tumor regression, even achieving pathological complete response. The assessment of tumor regression outcomes holds crucial significance for determining surgical approaches and establishing safe margins. Nonetheless, current research on tumor regression patterns remains limited, and there is considerable controversy surrounding the determination of a safe margin after neoadjuvant therapy. In light of these factors, this study aims to summarize the primary patterns of tumor regression observed following neoadjuvant therapy for rectal cancer, categorizing them into three types: tumor shrinkage, tumor fragmentation, and mucinous lake formation. Furthermore, a comparison will be made between gross and microscopic tumor regression, highlighting the asynchronous nature of regression in the two contexts. Additionally, this study will analyze the safety of non-surgical treatment in patients who achieve complete clinical response, elucidating the necessity of surgical intervention. Lastly, the study will investigate the optimal range for safe surgical resection margins and explore the concept of a safe margin distance post-neoadjuvant therapy.

## 1 Introduction

Colorectal cancer is a globally prevalent malignancy that poses a significant threat to human health (1). As a standard treatment approach for patients with locally advanced rectal cancer, neoadjuvant therapy has gained widespread utilization in the preoperative setting (2–4). The effectiveness of neoadjuvant therapy can result in diverse degrees and types of tumor regression, with implications for optimal surgical approaches and safe margin determination. Particularly in patients with locally advanced rectal cancer, the status of the safe margin plays a crucial role in preserving anal sphincter function and ensuring patients’ quality of life (5, 6). Consequently, this article seeks to provide a comprehensive review of tumor regression patterns and the appropriate safe margin distance following neoadjuvant therapy in rectal cancer. By deepening our understanding of these research advancements, we can enhance the development of personalized treatment plans and improve both the success rate and quality of life for patients.

## 2 The tumor regression patterns after neoadjuvant therapy for rectal cancer

After neoadjuvant therapy for rectal cancer, the tumor regression patterns manifest a complex and diverse array, characterized by the coexistence of various distinct patterns. According to current research, the primary tumor regression patterns comprise tumor shrinkage, fragmentation, as well as the formation of mucinous lakes.

### 2.1 Tumor shrinkage

This pattern denotes the overarching reduction in tumor size and infiltration, primarily along the mucosal direction, which serves as the primary response pattern for tumor regression and a key indicator for preoperative assessment of neoadjuvant therapy effectiveness (7, 8). Studies have demonstrated that neoadjuvant therapy leads to an average tumor size decrease of approximately 50%, with variable degrees of infiltration attenuation, including downstaging effects on the T stage, observed in certain patients (9). Downstaging holds independent prognostic significance for both disease-free survival and overall survival. This regression pattern forms the cornerstone of the “watch and wait” (W&W) treatment strategy post-neoadjuvant therapy, with imaging and endoscopic examinations serving as effective means of evaluating its efficacy.

### 2.2 Tumor fragmentation

This phenomenon encompasses the dissolution of the primary tumor mass, culminating in the development of small clusters of tumor cells. A prior study has revealed that approximately 80% of patients may undergo tumor fragmentation in response to neoadjuvant therapy. This fragmentation can yield asynchronous macroscopic and microscopic tumor regression. Several investigations have suggested that fragments can be detected in all directions (proximal, distal, and lateral) within a 3 cm range from the periphery of the gross residual tumor (lesion or scar). Tumor fragmentation correlates with residual lymph node metastasis, positive margins, and an unfavorable prognosis. Furthermore, isolated fragmentation alone does not lead to a reduction in tumor stage. Therefore, when selecting surgical approaches and determining the distant margin, careful attention should be paid to this regression pattern, ensuring the comprehensive elimination of tumor cells situated beyond the residual tumor mass. Another study indicated that inappropriate surgical strategies may readily result in tumor recurrence in cases involving tumor fragmentation.

### 2.3 Mucinous lake formation

Certain patients may exhibit the formation of mucinous lakes, also referred to as colloid reaction, in response to neoadjuvant therapy. A study conducted in 2015 unveiled that mucinous lakes can be observed in 40.7% of postoperative pathological specimens, with 26.0% containing mucinous lakes containing tumor cells and 14.7% consisting of mucinous lakes without tumor cells. Mucinous lakes harboring tumor cells are linked to a poorer prognosis; however, postoperative treatment effectively reduces the risk of recurrence. In a study with an average follow-up period of 79.0 months, patients without tumor cells in mucinous lakes exhibited 5-year and 10-year disease-free survival rates of 81.5% and 78.1%, respectively. Conversely, for patients with tumor cells in mucinous lakes, the rates were 63.7% and 61.2%, respectively (P=0.026). The presence of tumor cells in mucinous lakes resulted in a reduction of the 5-year and 10-year disease-free survival rates by 17.8% and 16.9%, respectively. These findings underscore the significance of completely excising residual tumor cells in mucinous lakes during surgery to minimize the likelihood of tumor recurrence. Presently, mucinous lakes without tumor cells are regarded as a tumor response rather than residual tumor. The team from Pakistan demonstrated that among patients who achieved pathological complete response (pCR), 27% had mucinous lakes without tumor cells, and this type of mucinous lake did not influence prognosis.

## 3 The difference between macroscopic regression and microscopic regression of tumors

A study conducted in Spain (10) revealed the predominant histopathological patterns of regression to be fibrosis (93.4%), colloid degeneration (29.2%), and cellular degeneration (64.2%). These findings corroborate the previously mentioned types of tumor regression. Notably, diverse neoadjuvant treatment regimens yielded significant variations in treatment outcomes for patients with distinct tumor types, although the majority of tumors displayed varying degrees of regression. Discrepancies between macroscopic regression and microscopic regression are frequently observed, often characterized by distant intramural dissemination—the phenomenon where a distance exists between the macroscopic tumor margin following neoadjuvant treatment and the microscopic tumor margin. This occurrence may be attributed to the fact that fragmented tumor cells (clusters) are only visible under microscopic examination and not discernible to the naked eye, thereby suggesting the persistence of residual tumor cells post-fragmentation. Additionally, it is plausible that prior to neoadjuvant therapy, tumor cells had already infiltrated beneath the normal mucosa and were incompletely eradicated by the treatment, subsequently reappearing under microscopic examination. Moreover, new small lesions may arise during neoadjuvant therapy. In a study comprising 20 patients with rectal cancer who underwent neoadjuvant therapy (2), microscopic observation revealed no residual tumor cells in 2 cases (10%), tumor cells confined within the macroscopic residual tumor boundary in 7 cases (35%), and distant intramural dissemination in the remaining cases. Among the latter, 50% exhibited a dissemination distance within 1 cm, while only 5% had a dissemination distance exceeding 1 cm. Similar findings demonstrated that 17.9% of rectal cancer patients exhibited no residual tumor cells microscopically after neoadjuvant therapy, with 23.2% showing tumor cells confined within the macroscopic residual tumor boundary, 39.3% displaying a dissemination distance within ≤0.5 cm, 16.1% with a dissemination distance of 0.5-1.0 cm, and only 3.6% demonstrating a dissemination distance >1 cm (11). Another study (12) illustrated that 71% of patients experienced distant intramural dissemination, with varying extents observed across different stages post-neoadjuvant therapy. The median dissemination distances and maximum dissemination distances for ypT1 were 0.0 mm and 4 mm, respectively; for ypT2, they were 2.5 mm and 9.0 mm, respectively; and for ypT3, they were 4.0 mm and 9.0 mm, respectively. These findings suggest that higher tumor stages may result in greater dissemination distances following neoadjuvant therapy.

Besides, evaluating the N stage of rectal cancer patients after nCRT holds significant clinical and research implications. Heijnen, et al. (13) indicated that patients with positive nodal stage at histology (ypN+) had significantly larger lymph nodes, both before and after CRT, than patients with negative pathologic nodal stage (ypN0). The total number of nodes per patient before and after CRT was not significantly different between ypN+ patients and ypN0 patients.

## 4 The necessity of surgical treatment after clinical complete response

Complete clinical response (CCR) refers to the complete disappearance of detectable cancer signs and symptoms in a patient following treatment. This term is commonly used in oncology to describe a situation where all evidence of cancer, as determined by physical examination, imaging studies, and laboratory tests, is no longer present after treatment (14, 15). Pathological complete response (pCR) refers to the absence of any detectable cancer cells in the surgical specimen after treatment. It is determined through a pathological examination of the tissue removed during surgery. Achieving pCR is considered a significant milestone in cancer treatment, indicating a complete eradication of cancer cells in the treated area (16). Renehan, et al. (17) reported that a total of 259 patients were enrolled, among whom 31 exhibited clinical complete response and were managed through the implementation of the “Watch & Wait (WW)” strategy. The adoption of this approach is integral in mitigating the need for superfluous treatments, thereby enabling patients to preserve their daily routines, minimizing financial constraints, reducing the associated treatment risks, as well as facilitating the formulation of bespoke treatment regimens. van der Valk, et al. (18) showed that in a cohort comprising 880 patients, the 2-year cumulative occurrence rate of local regrowth stood at 25.2%, with 97% of instances being localized within the bowel wall. Smith, et al. (19) indicated that the overall survival rate was determined to be 73% in the “Watch & Wait” (W&W) group, whereas that of the pathological complete response (pCR) group was 94%. Additionally, the disease-free survival rate in the W&W group was 75%, compared to 92% in the pCR group. Meanwhile, the disease-specific survival rate for the W&W group and the pCR group were 90% and 98%, respectively. Notably, patients in the WW group who exhibited instances of local regrowth showed a higher incidence of distant metastasis relative to those who did not experience such a complication. The W&W strategy may pose some risks in certain cases. For some cancer patients, although clinical assessment shows partial or complete remission (cCR), if there is a local recurrence of the tumor, this may indicate a risk of distant metastasis for the patient. Therefore, even when the W&W strategy is adopted, regular follow-up and examination are still crucial to timely adjust the treatment plan and avoid missing the optimal timing for treatment.

However, caution should be taken as a proportion of patients entering a W&W protocol will go on to develop a local regrowth of the primary tumor and therefore will require surgical resection. This means not all patients with a CCR will avoid surgery. Even though functional outcomes among patients undergoing W&W are clearly and far better than TME or even local excision (20), function may ultimately not be as perfect as one would expect or hope (21). Interesting data suggest that functional outcomes of patients undergoing W&W are not necessarily perfect, possibly due to the effects of radiation therapy to the rectum and anal sphincters (22). Hupkens, et al. (20) reported that following a successful implementation of the watch-and-wait approach, patients reported a superior quality of life across multiple domains in comparison to those who underwent chemoradiation therapy and surgery. Nonetheless, it is important to acknowledge that chemoradiation therapy carries its own long-term side effects. Notably, one-third of patients who underwent the watch-and-wait strategy experienced significant symptoms associated with low anterior resection syndrome, while this percentage rose to 66.7% within the total mesorectal excision group. And Quezada-Diaz, et al. (21) indicated that the comparison suggesting potentially superior bowel function among WW patients in contrast to those undergoing TME should be approached judiciously. This assessment is tempered by the exclusion of surgical complications from consideration and the predominance of patients characterized by a low risk profile for sphincter damage resulting from radiation and surgical interventions. Therefore, when deciding whether to proceed with surgical treatment, it is essential to consider the overall condition of the patient, including factors such as age, physical health, and tumor characteristics. In general, there is currently no consensus regarding the necessity of surgical intervention after clinical remission in rectal cancer patients. Individualized discussions and decisions are required for each patient to strike a balance between the benefits and risks of treatment. In the future, further clinical research is needed to clarify this issue and guide clinical practice.

## 5 Rectal cancer safe distance of distal margin after neoadjuvant therapy

Neoadjuvant therapy is increasingly employed in colorectal cancer patients; however, the lack of a standardized criterion for determining the safe distance of the distal margin after neoadjuvant therapy has led to controversy and uncertainty in clinical practice. Furthermore, limited research exists regarding the association between the distance of the distal margin post-therapy and tumor recurrence, metastasis, and postoperative complications. In patients without neoadjuvant therapy, the distal margin serves as an indicator for determining the safe distance. However, neoadjuvant therapy often reduces tumor volume, potentially resulting in excessive removal of normal tissue, particularly in low rectal cancer patients. For middle and high rectal cancer patients, wider resectable distal margins are feasible. Nevertheless, maintaining an adequate amount of residual rectal tissue is crucial in low rectal cancer patients to preserve anal function and enhance quality of life. Surgical principles dictate minimizing the distance of the distal margin while ensuring a low postoperative recurrence rate. As neoadjuvant therapy becomes more prevalent, radical surgical treatment is typically required for treated patients. However, the absence of uniform resection standards often leads surgeons to rely on personal experience to determine the distance of the distal margin. Disparities in rectal cancer surgery diagnosis and treatment capabilities exist among different regions, hospitals, and even individual surgeons in China. While tertiary hospitals in developed areas possess extensive surgical experience, grassroots hospitals may face limitations due to resource and technical constraints when determining the safe distance of the distal margin. Ensuring consistency in the safe distance across hospitals and healthcare professionals at all levels is therefore a pressing issue. To guide clinical practice effectively, research on the safe distance of the distal margin post-therapy needs to be enhanced, and standardized criteria and guidelines should be established. Concurrently, resource allocation and medical training must be strengthened to ensure high-quality rectal cancer surgery and improve patient quality of life. Future research should further explore the relationship between the distance of the distal margin post-therapy and tumor recurrence, metastasis, and postoperative complications, providing a more reliable basis for clinical decision-making.

Presently, certain perspectives propose that a 1 cm margin at the distal resection edge following neoadjuvant therapy is relatively safe. In patients who underwent neoadjuvant therapy, the rate of pathologic complete response (pCR) was 17.8%, and 58.9% of patients had intramural spread at the distal wall, with a mean distance of 0.56 ± 0.3 cm (range 0.2-1.8 cm). Among these patients, 87.9% had a spread distance <1 cm. Therefore, studies suggest that a 1 cm distal margin may be sufficient for the majority of patients (11). In another study (23), 88 patients with locally advanced rectal cancer were examined, and it was found that the 5-year overall survival rates for patients with a distal margin <1 cm and ≥1 cm were 93.2% and 95.7%, respectively (P=0.642). Additionally, the 5-year local recurrence-free survival rates were 92.3% and 93.4%, respectively (P=0.936). The study concluded that even with a distal margin <1 cm, R0 resection could achieve favorable outcomes in low rectal cancer after neoadjuvant therapy. Reducing the distal margin distance might allow surgeons to preserve the anal sphincter without compromising local recurrence-free survival and overall survival. Similarly, research has demonstrated that a distal margin <1 cm is associated with local recurrence in patients with locally advanced rectal cancer who receive preoperative neoadjuvant therapy. This association is particularly evident in patients with ypT2-T4 tumors, and more than half of the local recurrences occur in the central region, such as the presacral area and anastomotic site. In the ypT2-T4 group, the cumulative incidence of recurrence within 3 years was 2.3% in the ≥1 cm margin group and 9.8% in the <1 cm margin group (P=0.001). In contrast, a distal margin <1 cm is not a significant risk factor for local recurrence in patients with ypT0-T1 tumors (24). This difference may be attributed to the varying effectiveness of neoadjuvant therapy in different patients and the extent of tumor spread at different stages. Studies have shown that as the tumor stage increases, the extent of distal spread also increases (12). For ypT0-T1 patients, neoadjuvant therapy has relatively better efficacy, with minimal intramural spread at ypT1 stage. However, for ypT2-T4 patients, neoadjuvant therapy does not achieve ideal results, resulting in significantly greater spread distance than ypT1 stage. On the other hand, some viewpoints suggest that as long as all margins (distal, proximal, and circumferential) are free of residual cancer cells after neoadjuvant therapy, the distance of the distal margin does not have a statistically significant impact on local recurrence and long-term survival (25). Relevant data shows that over 50% of patients have intramural spread after neoadjuvant therapy, with tumor cells spreading unevenly and unpredictably (6). However, the majority of patients have a distal spread distance within 1 cm, and few patients have tumor spread distances exceeding 2 cm (12). Therefore, a 1 cm distal margin distance may already be considered safe in this regard. Research also indicates that patients with a distance exceeding 2 cm, even if all margins are negative after surgery, have a poor long-term prognosis (26). This suggests that for such patients, a distal margin above 2 cm may not have practical clinical significance in terms of prognosis.

The physiological and psychological impact of rectal cancer on patients’ anal function should not be underestimated. The primary goal of rectal cancer surgery is to achieve complete tumor removal while preserving anal organ and function to the greatest extent possible. This objective is particularly critical in the treatment of patients with low or ultra-low rectal cancer, where the distance from the resection edge plays a crucial role in determining the surgical approach. Therefore, a comprehensive assessment of the patient’s tumor characteristics is imperative prior to surgery, with a specific focus on understanding the extent of tumor regression and determining the appropriate resection margin in a scientifically and rational manner (27). In addition to traditional imaging examinations, the utilization of rectal intracavitary ultrasound and full-spectrum multipoint colonoscopy pathology biopsy is indispensable for a more accurate evaluation of the tumor condition. These methods provide detailed information, including the depth of tumor infiltration and the extent of diffusion within the distal wall. For patients with significant tumor regression and small residual tumors undergoing procedures such as rectal anterior resection, accurately evaluating the intraoperative resection margin within the intestinal cavity can be challenging. To address this issue, preoperative tumor staining and titanium clip positioning have become effective means to facilitate precise observation and judgment of the resection margin during surgery. Performing rapid pathological examination during surgery is vital to prevent positive resection margins. If residual tumor cells are detected at the resection margin, it is crucial to promptly adjust the surgical plan, increase the resection margin distance, and ensure complete tumor removal. Accurate pathological evaluation plays a decisive role in this process. Furthermore, in terms of the choice of surgical approach, transanal total mesorectal excision (taTME) has gained recognition as a highly esteemed method. Compared to traditional laparoscopic total mesorectal excision, taTME offers the advantage of directly visualizing the resection margin, which is particularly beneficial for male patients with pelvic stenosis, rectal mesentery hypertrophy, and obesity. This surgical approach is expected to provide superior resections of higher quality and establish a solid foundation for postoperative recovery. For patients who maintain satisfactory anal function after neoadjuvant therapy, intersphincteric resection (ISR) represents a viable surgical option. ISR not only enables the evaluation of the resection margin under direct visualization but also achieves ultra-low preservation of the anus. It embodies an ideal surgical approach that combines high-quality tumor removal with functional preservation (28). Overall, the surgical decision-making process for rectal cancer patients necessitates a comprehensive consideration of individual differences, tumor characteristics, and postoperative quality of life. By effectively utilizing advanced medical technology in a comprehensive manner, personalized surgical plans can be developed to maximize anal function preservation and enhance the patient’s postoperative quality of life. In this process, the collaboration of a multidisciplinary team and interdisciplinary comprehensive evaluation will yield improved treatment outcomes.

## 6 The management of lateral pelvic lymph node (LPN) metastasis in for rectal cancer

Currently, a contentious debate persists between Japan and Western nations regarding the appropriateness of neoadjuvant chemoradiotherapy (nCRT) as a substitute for lymph node dissection (LPND) in the management of LPN metastasis. The Japan Society for Cancer of the Colon and Rectum advocates for the integration of TME with LPND for the treatment of stage II and III middle-to-lower rectal cancer (29). However, the Japanese Clinical Oncology Group has noted a pathologic positivity rate of only 7% for LPNs post-surgery, indicating potential shortcomings in patient selection and surgical criteria, leading to overtreatment (30). Simultaneously, an expanding corpus of literature substantiates that nCRT alone proves ineffective in achieving complete eradication of metastatic LPNs without concomitant LPND, consequently posing a heightened risk of recurrence with both approaches (4, 31).

In recent times, there has been a notable acknowledgment and backing for the treatment paradigm involving targeted LPND subsequent to nCRT. Utilizing 7mm or 8mm as the diagnostic threshold for presumed LPN metastasis has gained traction, with evidence suggesting that administering nCRT prior to LPND does not yield substantial enhancements in local disease management or overall survival rates. For example, Yang et al. (32) showed that a cohort of 77 consecutive patients who underwent TME and lateral lymph node (LLN) dissection was analyzed. Among them, 22 individuals (28.6%) were diagnosed with pathological positive lateral node metastasis. It was found that 47 patients (61%) opted for nCRT as part of their treatment plan. Notably, the study identified the pretreatment maximum diameters of LLN (≥ 8 mm) as independent risk factors for LLN metastasis. And Kawai et al. (33) found that the estimated incidence of lLLN metastasis following chemoradiotherapy was 9.3%. Despite a high tendency for distant recurrence in patients with lLLN metastasis, 40.4% of them achieved a recurrence-free survival of over 5 years. Their analysis of lLLN sizes identified a lLLN size of ≥8 mm before chemoradiotherapy as the optimal criterion for lLLN dissection, exhibiting a sensitivity and specificity of 92.3% and 78.7%, respectively. Meanwhile, Zhou et al. (34) demonstrated the safety and feasibility of administering nCRT prior to TME + LPND, with low mortality rates and acceptable morbidity. They also highlighted that post-nCRT LPN sizes of ≥7 mm were independent predictive factors for pathological LPNM after nCRT in rectal cancer patients with clinical LPNM, suggesting that patients with these characteristics should consider LPND post-nCRT. Furthermore, Ogura et al. (35) revealed that CRT plus TME plus lLLN dissection resulted in a 5-year lateral local recurrence rate of 5.7%, significantly lower than that observed in patients who underwent CRT plus TME with LLNs measuring at least 7 mm. In conclusion, LPND without nCRT proves effective and adequate in preventing local recurrence in patients with LPN metastases, warranting further randomized controlled trials for validation.

## 7 The mechanism of distant metastasis in patients with local recurrence

There are several possible underlying mechanisms related to the high rate of distant metastasis in rectal cancer patients with local recurrence.(1) Tumor cell invasion ability: The invasion and metastasis ability of tumor cells is an important factor in the distant metastasis of rectal cancer patients. (2) Existence of micrometastases: micrometastases refer to distant metastatic lesions with a size smaller than 1mm, which cannot be detected by current diagnostic methods due to their small size. (3) Surgical trauma and tumor implantation: Surgical treatment may result in surgical trauma and implantation of tumor cells in the operative area. These implanted tumor cells may proliferate after local recurrence and ultimately lead to distant metastasis. (4) Immune suppression: Local recurrence may lead to sustained suppression of local immune responses, making tumor cells more likely to evade immune surveillance and form metastatic lesions in distant sites. (5) Angiogenesis and Inflammatory reactions: Local recurrence can cause changes in the tumor microenvironment, such as promoting angiogenesis and inflammatory reactions. Angiogenesis provides oxygen and nutrients to tumors, while also serving as a pathway for the distant metastasis of tumor cells. Inflammatory reactions may promote the invasion and migration of tumor cells, accelerating tumor progression and metastasis.

## 8 Outlook

There is a vast body of literature on the safe distance from the resection margin, yet some studies exhibit significant differences in tumor T staging within the groups, leading to substantial bias. A small number of studies have inconsistent treatment standards, with only a fraction of patients receiving adjuvant chemotherapy, thereby compromising the reliability of the experiments. Additionally, the neoadjuvant therapy regimen significantly impacts the patterns and extent of tumor regression, thus affecting the safe distance from the resection margin. There is currently no unified standard for the safe distance from the resection margin following neoadjuvant therapy for rectal cancer. However, existing research results show that a 1 cm surgical resection margin can indeed eliminate the vast majority of tumor cells in most patients. In the future, based on current standard treatment protocols, randomized controlled studies should be conducted to classify patients at different stages following neoadjuvant therapy for rectal cancer and determine the appropriate safe distance from the resection margin. Consistent treatment standards and larger-scale research can improve the surgical precision and treatment outcomes following neoadjuvant therapy for rectal cancer.

## Author contributions

GY: Investigation, Validation, Writing – review & editing. HC: Writing – original draft. GZ: Conceptualization, Writing – review & editing. YW: Conceptualization, Writing – review & editing.
